# The Foreign Journals: Pathology, Practical Medicine, and Therapeutics

**Published:** 1841-04

**Authors:** 


### PATHOLOGY, PRACTICAL MEDICINE, AND THERAPEUTICS.

### *Observations and Experiments on the Employment of Platina in Medicine.* By M. FERD. HOEFER, M.D.

THIS is a lengthy but valuable contribution to medical therapeutics. The author first treats of platina in the metallic state, and of its principal compounds, and then examines its physiological action by experiments on animals, and on man in a state of health, and when suffering from disease. Several cases are given to illustrate the statements of the author; but our space prevents us giving more than his general conclusions, which are as follows:

1. The preparations of platina (chlorides) are poisons; the perchlorides in the dose of a scruple; the double chloride of platina and sodium in the dose of two scruples.

2. The chlorides of platina, (perchloride and double chloride of platina and sodium,) are less dangerous than the salts of gold, and the corrosive sublimate.

3. The perchloride of platinum in concentrated solution, produces acute itching on the skin, followed by a cutaneous eruption in the situation where the solution has been applied. Taken internally, it at first irritates the mucous membrane of the stomach, occasions cephalalgia, reacts on the nervous centres, and thus exercises a particular alterative action on the fluids of the economy.

4. The double chloride of platina and sodium does not produce local irritation on the skin. Taken internally, it does not react on the nervous centres in a manner so sensible as the simple perchloride. It more particularly augments the urinary secretion.

5. The perchloride of platinum is a remedy very efficacious in the treatment of syphilitic diseases; and particularly of those which are old, inveterate (constitutional).

6. The double chloride of platina and sodium is well suited to the treatment of recent or primary syphilitic diseases. It is also very efficacious in the treatment of rheumatic affections.

7. Platina appears to rank in the class of medicines called alteratives, with gold, iodine, and arsenic. It differs from mercury in acting after a previous excitation, and as its administration does not entail any of the evils with which mercury is charged. The salts of gold, which appear to be poisonous in much smaller doses than the salts of platina, are, according to writers, only efficacious in certain cases of constitutional syphilis.

8. Platina, as an alterative medicine, is preferable to mercury and to gold.

*Gazette Médicale de Paris.* 28 *Nov.* 1840.

*Complete Paralysis of the fifth pair on one side, with complete Abolition of Sight, Hearing, Smelling, and Taste on the same side, cured by Galvanism.* By M. James, Interne at La Charité.

At the sitting of the Royal Academy of Medicine on the 21st of October, 1840, M. James presented a young man who had been affected for two years with paralysis of the fifth pair on one side, which had been cured by the aid of galvanism.

The patient was in the wards of M. Andral, and stated that after neuralgia of the side of the face corresponding to the paralysis, which lasted eight days, the paralysis supervened. On his admission there was absolute loss of sensibility of the whole of the skin receiving the three sensitive branches of the fifth pair. It was proved that the conjunctiva, the nasal and lingual mucous membrane, and that of the cavity of the tympanum on the affected side, had no tactile sensibility. The special sensibilities of these four organs were also completely abolished on the affected side. M. James was convinced that the base of the tongue did not perceive tastes on the affected side, and was not sensible of the contact of a body. The muscles receiving the nerves of the motor branch were also paralysed, as proved by the unequal depression of the jaw, with an evident displacement outwards on the paralysed side, and impossibility of closing the jaws on that side. All these functional disorders yielded to galvanism applied, in a considerable number of sittings, to the different points affected. The organs of sense first recovered their sensibility; the tactile sensibility returned afterwards.

*Gazette Médicale de Paris. Octobre* 24, 1840.

---

*On the Employment of the Sulphate of Alumina in the Ulcerations and Inflammations of Mucous Membranes.* By M. Delmas.

The author of this paper concludes, from numerous observations, that alum dissipates the inflammation of mucous membranes ;and that, applied to solutions of continuity of these membranes, it accelerates their cicatrization, while its action is never injurious when carefully applied. It also rapidly accelerates the cicatrization of cutaneous ulcerations, though in this case it is apt to induce pain and reaction. In syphilitic ulcers it is a most valuable topical application.

*Bulletin Général de Thérapeutique,* 15 *et* 30 *Novembre,* 1840.

---

*Fibres in the walls of the Gall-Bladder.* By M. Barth.

M. Barth has presented to the Anatomical Society of Paris, a dilated gall-bladder, the walls of which offered very apparent fibres, interlaced, and having a great resemblance to muscular fibres.

*L'Expérience.* 19 *Novembre,* 1840.

---

*On Acephalocysts in the Brain.* By Dr. Michéa.

Acephalocysts are so rarely found in the brain, that we record a notice of two cases which we observed in the practice of M. Martin Solon. The symptoms in this first case, in a man 53 years of age, resembled those of sanguineous effusion into the right hemisphere of the brain, followed by hemiplegia. The dura mater was found very vascular, with slight serous effusion into the arachnoid cavity, and injection of the pia mater. Opaline vesicles were found in both hemispheres, but mostly in the anterior lobe of the left, of the size of large peas, containing a diaphanous liquid, the centre of which was occupied by a small, globular, opaque milky-white body, without head or tail visible by the microscope. The second case was an epileptic patient, aged twenty-three, in whom the acephalocysts were found in smaller numbers.

*Gazette Médicale de Paris.* 21 *Novembre,* 1840.

